# Progressive Irreversible Proprioceptive Piezo2 Channelopathy-Induced Lost Forced Peripheral Oscillatory Synchronization to the Hippocampal Oscillator May Explain the Onset of Amyotrophic Lateral Sclerosis Pathomechanism

**DOI:** 10.3390/cells13060492

**Published:** 2024-03-12

**Authors:** Balázs Sonkodi

**Affiliations:** 1 Department of Health Sciences and Sport Medicine, Hungarian University of Sports Science, 1123 Budapest, Hungary; bsonkodi@gmail.com; 2 Department of Sports Medicine, Semmelweis University, 1122 Budapest, Hungary

**Keywords:** amyotrophic lateral sclerosis, Piezo2, syndecan-3, proton-based ultrafast signaling, MyoD-family inhibitor proteins

## Abstract

Amyotrophic lateral sclerosis (ALS) is a mysterious lethal multisystem neurodegenerative disease that gradually leads to the progressive loss of motor neurons. A recent non-contact dying-back injury mechanism theory for ALS proposed that the primary damage is an acquired irreversible intrafusal proprioceptive terminal Piezo2 channelopathy with underlying genetic and environmental risk factors. Underpinning this is the theory that excessively prolonged proprioceptive mechanotransduction under allostasis may induce dysfunctionality in mitochondria, leading to Piezo2 channelopathy. This microinjury is suggested to provide one gateway from physiology to pathophysiology. The chronic, but not irreversible, form of this Piezo2 channelopathy is implicated in many diseases with unknown etiology. Dry eye disease is one of them where replenishing synthetic proteoglycans promote nerve regeneration. Syndecans, especially syndecan-3, are proposed as the first critical link in this hierarchical ordered depletory pathomechanism as proton-collecting/distributing antennas; hence, they may play a role in ALS pathomechanism onset. Even more importantly, the shedding or charge-altering variants of Syndecan-3 may contribute to the Piezo2 channelopathy-induced disruption of the Piezo2-initiated proton-based ultrafast long-range signaling through VGLUT1 and VGLUT2. Thus, these alterations may not only cause disruption to ultrafast signaling to the hippocampus in conscious proprioception, but could disrupt the ultrafast proprioceptive signaling feedback to the motoneurons. Correspondingly, an inert Piezo2-initiated proton-based ultrafast signaled proprioceptive skeletal system is coming to light that is suggested to be progressively lost in ALS. In addition, the lost functional link of the MyoD family of inhibitor proteins, as auxiliary subunits of Piezo2, may not only contribute to the theorized acquired Piezo2 channelopathy, but may explain how these microinjured ion channels evolve to be principal transcription activators.

## 1. Introduction

Amyotrophic lateral sclerosis (ALS) is a multisystem lethal neurodegenerative disease, with an enigmatic progressive loss of motoneurons and aging-associated evolvement [1]. In spite of the fact that ALS has been identified for more than 100 years, the exact location of ALS onset is still not known and the consequent pathophysiology is far from entirely understood.

The prevalence of sporadic ALS cases is close to 90% [2], and the identified familial cases represent only about 10% [3]. Nevertheless, the genetic background of ALS is still quite obscure, because the heritability of sporadic patients is estimated to be as high as 50% [4]. Accordingly, the contribution of long non-coding DNA and RNA is linked to the ALS disease process [5,6,7], and this is how, for example, the dysregulation of piRNA/PIWI proteins could have a role in ALS pathogenesis [8]. The majority of the genes associated with ALS are either susceptibility factors or genes linked to other neurodegenerative conditions; hence, they have no direct causal relationship with ALS.

Somatosensory involvement and sensory circuit dysfunction in ALS disease process are evident in the presymptomatic stage [9,10,11,12]. According to a new non-contact dying-back injury mechanism theory for ALS, the primary damage is an acquired irreversible proprioceptive terminal Piezo2 channelopathy in the muscle spindles [13,14]. This Piezo2 channelopathy is proposed to be a principal transcription activator [14]; hence, it could reveal the underlying genetic variants that become more apparent during the time window of this ion channel microinjury. Moreover, this acquired irreversible Piezo2 channelopathy is also suggested to miswire proprioception and dysregulate the primary pain pathways traveling through the dorsal horn of the spinal cord in ALS patinets [14]. The proposed underpinnings of these alterations are the lost imbalanced subthreshold Ca^2+^ currents, N-methyl-d-aspartate (NMDA) activation, and the resultant lost L-type Ca^2+^ currents that induced lost activation in wide dynamic range (WDR) neurons [14]. Indeed, likely pathogenic variants of Ca_v_1.3 and Na_v_1.1 ion channel encoding genes were detected in ALS in support of the Piezo2 channelopathy theory [15]. An interesting recent finding showed dysfunctionality and successive disruptions in the postsynaptic structure of the neuromuscular junctions in ALS [16]. The current author suggests that this finding could be the consequence of the theorized proprioceptive terminal Piezo2 channelopathy and the resultant proprioceptive miswiring and VGLUT1/Ia synaptic disconnection in motoneurons [14,17,18].

Another proposed consequence of the Piezo2 channelopathy theory is that it disrupts the long-suspected Piezo2–Piezo1 cross-talk in ALS [14]. Syndecans are suggested as the first-line critical mediators of this Piezo2–Piezo1 crosstalk [17,19]. Every cell accommodates at least one syndecan, but they also exhibit redundancy in order to compensate for each other [20]. These transmembrane proteoglycans have four family members, and syndecan-3 is the largest amongst them. The dense representation of syndecan-3 in the central nervous system (CNS) is a well-known fact [21]. Lately the relevance of their presence and functionality on the periphery is emerging [21]. The actin-dependent central role of syndecan-3 in cell adhesion, migration, and neurite outgrowth in neurons is also known [22]. Correspondingly, their transmembrane localization serves as one crucial link between the actin cytoskeleton and the extracellular matrix. In addition, syndecan-3 is a functional player in satiety control and spatial memory encoding [21,23], but is also involved in inflammation and angiogenesis in certain diseases [21].

An important finding is that nerve injury to primary afferents upregulates syndecan-1 expression in the dorsal root ganglion (DRG) [24]. However, this has been proposed as a compensatory mechanism in response to autogenic syndecan-3 depletion or functional alteration, since syndecan-1 and syndecan-3 have redundant features [19]. Interestingly, syndecan-3 is implicated in the pathology of Alzheimer’s disease [25], but no relationship to ALS has been reported yet. It is important to remark that the reanalysis of the potential pathogenic gene variants from a previous ALS study confirmed the absence of pathogenic variants of Piezo2 and Piezo1 [15]. This exclusion substantiated the idea that the suggested irreversible Piezo2 channelopathy is acquired and not inherited, as was theorized in the non-contact dying-back injury mechanism theory for ALS [15]. Nevertheless, the current opinion piece theorizes that shedding or charge altering variants of the syndecan-3-encoding *SDC3* gene may contribute to how the acquired irreversible Piezo2 channelopathy could evolve during the aging process, leading to the progressively lost remodeling and regeneration of the affected muscles in ALS.

## 2. Piezo2 Channelopathy, Piezo2–Piezo1 Crosstalk, and Syndecans

The aforementioned theory postulates that ALS is initiated via acquired irreversible proprioceptive terminal Piezo2 channelopathies, leading to the progressive non-contact dying-back injury mechanism of this lethal disease [1]. According to the acute form of this Piezo2 channelopathy, suggested in delayed onset muscle soreness (DOMS) [18,26], the intrafusal primary afferents with Piezo2 in their terminals could be microdamaged in an autogenic way under an acute stress response [26]. This acquired channelopathy may also mean an impaired crosstalk between Piezo2 and Piezo1 channels within the affected compartment [27]. Syndecans, especially syndecan-3 on intrafusal proprioceptive neurons, are proposed as a critical player in this Piezo cross-communication [17,19]. Thus, syndecan-3 could bear relevance in regard to the theoretical loss of Piezo2–Piezo1 crosstalk in ALS pathomechanism onset.

It is important to note that DOMS is viewed as a dichotomous injury mechanism [28,29]. The primary damage of this bi-phasic injury has been proposed to be the Piezo2 channelopathy on the intrafusal proprioceptive terminal, and the secondary damage is a harsher tissue injury in the extrafusal space [18]. Conclusively, DOMS is also suggested to be a bi-compartmental injury, embracing the intra- and extrafusal space, as well [18]. Moreover, DOMS may involve all four muscle sensory afferents: namely, the Type Ia and Type II fibers in the intrafusal space, and Type III and Type IV fibers in the extrafusal space [30]. It has been shown that glial cell-line-derived neurotrophic factor (GDNF) upregulation by cyclooxygenase-2 (COX-2) is essential in DOMS [31]. It is also known that GDNF directly interacts with syndecan-3, acting like a novel receptor for it [32]. Consequently, the syndecan-3-containing Type III sensory terminals in the extrafusal space could be blunted by GDNF under hyperexcitation, hence paving the way for neuroinflammation. Syndecan-3 also facilitates neurite outgrowth [32]. Furthermore, GDNF interacts with nerve growth factor (NGF) in the extrafusal muscle space [33], and NGF sensitizes Type IV sensory neurons through COX-2 in DOMS with the contribution of bradykinin [34]. These cross-talks were indeed theorized in the acute compression axonopathy theory for DOMS [13,30].

Glutamate is suggested to sensitize Type Ia sensory neurons in the intrafusal space [13], as an elevated glutamate level is recognized in DOMS [35]. Furthermore, the theory of Piezo2 channelopathy also entails the impairment of the glutamate vesicular release machinery [26]. It is important to note that syndecans-3 may have a role in the control of this vesicular trafficking [36]. In addition, prolonged mechanotransduction is proposed to induce syndecan clustering and shedding on the extracellular membrane surface in unconventional lipid and cholesterol rafts [36]. According to the Piezo2 channelopathy theory, the membrane lipids and cholesterol surrounding Piezo2 could be depleted during excessively prolonged mechanostransduction under allostasis [18,28]. It is indicative of the acquired Piezo2 channelopathy theory and the functional relevance of lipids that the replacement of linoleic acid improves Piezo2 function in a neurodegenerative disease [37]. It is worthy of note that lipid and cholesterol dyshomeostasis has an often-reported association with ALS, although a controversial one [38]. Nevertheless, it has not been investigated in the intrafusal compartment.

Moreover, a recent finding showed that atypical glutamate receptors on primary afferent terminals with glutamate vesicular release controls stretch sensitivity [39]. Thus, this emptying of lipid and membrane cholesterol rafts may not only underpin the imbalanced regulation of NKT-like cells [40], but might contribute to the impaired glutamate vesicular release through lost trafficking control [13,41]. In line with this, the homeostasis of Interleukin-17 (IL-17)-producing NKT cells (NKT17) is negatively regulated by syndecan-1 [42]. Hence, the chronic depletion or functional alteration of syndecan-1 due to the lost Piezo2–Piezo1 crosstalk could be an explanation for the lost regulation of NKT cells. This is despite the high NKT cell production in ALS [43] that is suggested to be the consequence of the progressive, irreversible proprioceptive terminal Piezo2 channelopathies. Indeed, NKT17 cells and γδ T cells (Tγδ17) have SDC1 markers [42], and SDC3 is a paralog of the SDC1 gene. Thus, syndecan depletion or functional alteration not only could impair glutamate vesicular release and resultant glutamate excitotoxicity, but could alter the NKT cell immune defense in response to progressive irreversible Piezo2 channelopathy-induced increased NKT cell production in ALS.

It is known that carbonic anhydrase (CA) is upregulated under hypoxia-induced stress in the neurons of the brain [44]. Accordingly, this membrane-associated upregulation of CA1 is also present in the spinal cord in ALS, in addition to an altered distribution of the subpopulation of CA1 on the endoplasmic reticulum membranes of motor neurons [45]. This finding was unexpected, because the CA2 isoform is the one mostly available in the CNS [45]. Indeed, CA2, with its protein crystal structure, has an important signaling role in glutamatergic neuronal function [46]. Correspondingly, the current author suggests that CA protein crystals have a pivotal role in the proton-based cross signaling between motoneurons and syndecan-3 assisted Piezo2 mechanotransducing proprioceptive glutamatergic neurons. This signaling is likely through VGLUT1. Consequently, the proposed irreversible Piezo2 channelopathy may cause VGLUT1/Ia synaptic disconnection on motoneurons [14,18]. Moreover, the lost Piezo2 resonance on the proprioceptive afferents may also upregulate CA1 in the spinal cord of ALS. As a result, it could even alter the distribution of the subpopulation of CA1 on the endoplasmic reticulum membranes of motor neurons at the level of the affected spinal cord segment. It could be translated as a feed-forward compensatory protein crystal-based upregulation mechanism due to lost proton-based proprioceptive signaling.

This important observation remains valid when it comes to the substantially upregulated expression of Acid Sensing Ion Channel Subunit 2 (ASIC2) in motoneurons in SOD1 mice and humans with sporadic ALS [47]. This is in spite of the fact that their paper was retracted later for other reasons [46]. It is noteworthy that ASIC2 ion channels are present on proprioceptive Type Ia afferent terminals [48]. The ablation of ASIC2 will result in altered-muscle-spindle-derived stretch responses and motor coordination, in addition to impairment to spinal alignment [49]. This paper proposed that Piezo2 is needed for mechanostransductory signaling on proprioceptive neurons and ASIC2 modulates this signaling [49]. In support, a loss of function on Piezo2 also causes impaired proprioception [50] and scoliosis [51].

The above-suggested proprioceptive Piezo2 channelopathy may induce miswiring on motoneurons and the spinal dorsal horn [18], leading to the delay of the static phase firing encoding of the stretch reflex [41,52]. Accordingly, the theoretical irreversible functional loss of Piezo2 on proprioceptive terminals [41] and the resultant progressive loss of proprioceptive protection, not to mention acidosis, glutamate mishandling, and hypoxia, could contribute to the observed upregulated expression of ASIC2 and altered distribution of CA1 on the endoplasmic reticulum membranes of motor neurons in ALS. This proposed mechanism could result in the aforementioned recent finding: namely, the abnormal postsynaptic structure of the neuromuscular junction in ALS [16].

## 3. The Metabolic Switch

Earlier it was refuted that lactate has a role in the DOMS mechanism [53]. Notwithstanding, a recent opinion paper rationed that lactate may still relate to DOMS, primarily in the intrafusal space [54]. Correspondingly, lactate could activate ASIC3 channels on intrafusal Type II afferents [54], and probably ASIC2 on Type Ia afferents. In support, lactate could enter through selective barriers during exercise, like the blood–brain barrier [55]. The functional relevance of the selective barrier of the muscle spindle has been emphasized [30] and it might be such a gateway for lactate, as well. Moreover, it has also long been known that lactate–proton co-transport has a role in the acidification of the interstitial space under nervous tissue hypoxia [56]. Furthermore, lactate is capable of activating the mitochondrial electron transport chain [57]. The first damage phase, later coined as Piezo2 channelopathy [18,26], of the acute compression axonopathy theory for DOMS emphasized the contribution of the mitochondrial electron-transport-chain-generated free radicals in the intrafusal space [30]. The elevated level of reactive oxygen species is known to increase proton leak, but a higher level of proton leak limits the generation of reactive oxygen species in order to prevent functional damage to mitochondria [58,59]. Interestingly, both mitochondrial uncoupling protein-2 and Piezo2 increase the activation of endothelial nitric oxide synthase in support of the co-involvement of mictochondria and Piezo2 channels in this protective process [60,61]. However, this protective mechanism could derail under allostasis when mechanotransduction is prolonged excessively, and that is when Piezo2 channelopathy as a mechano-energetic microdamage may evolve [18,54]. The energetic part of this microdamage may also be associated with a metabolic switch [40,54]. In line with this, activated neurons have a preference for lactate over glucose as an energy source [62]. Moreover, the neuronal depolarization induced by circulating glucose was solely regulated by astrocyte-mediated lactate, hence demonstrating the existence of the astrocyte-neuron lactate shuttle [63]. An analogous lactate shuttle mechanism is suggested in the muscle spindle, as well [54]. However, under allostasis, when mechanotransduction is excessively prolonged, this lactate shuttle mechanism could be impaired, leading to Piezo2 channelopathy due to the referred metabolic switch [54]. Noteworthily, astrocytes, and satellite cells contain Piezo1, while somatosensory neurons, like proprioceptive ones, contain Piezo2. Moreover, Piezo2 channelopathy is suggested to disrupt Piezo2–Piezo1 crosstalk within a Piezo2 microinjured compartmental micromilieu. Piezo2 channelopathy is also theorized to be associated with the impairment of glutamate vesicular release [52], and protons are known to regulate VGLUT proteins [64]. These VGLUTs act like proton–glutamate antiports [65]; hence, the impairment of glutamate vesicular release leading to glutamate excitotoxicity may cause the aforementioned VGLUT synaptic disconnection.

The author proposes that the catalytically inactive CAs are functioning like “proton-collecting/distributing antennas” [66], as suggested in the case of syndecan-3 for proprioceptive neuron terminals [19]. These “antennas” shuttle protons along the lactate flux into neurons and buffer acute changes in pH during anoxic autogenic stress in the neural extracellular and interstitial space. Correspondingly, it is theorized in DOMS that excessively prolonged mechanotransduction on Type Ia afferent terminals under autogenic allostasis, induced by voluntary strenuous and/or unaccustomed eccentric contractions, may not only induce local anoxia/hypoxia in the compartmental extracellular micromilieu, but may also impair the lactate shuttle mechanism and ASIC2 proton handling [54]. Consequently, the suggested Piezo2 channelopathy may lead to excessive proton leak, glutamate excitotoxicity, acidosis, and intrafusal Type II afferent sensitization through ASIC3 [54].

Hence, this molecular-switch-induced impaired metabolic machinery could be present within the muscle spindle at the Type Ia proprioceptive terminals in cooperation with intrafusal satellite cells. This analogy is based on the astrocyte-neuron lactate shuttle shown in the CNS [54]. It is important to note again the role of Piezo1 in satellite cell function, especially in muscle regeneration [67]. Thus, the impairment to Piezo2–Piezo1 crosstalk, preceded by the aforementioned impaired lactate-shuttle-induced metabolic switch-derived satellite cell activation, could enhance Piezo1 control both intra- and extrafusally. However, the Piezo2–Piezo1 crosstalk is suggested to be progressively blunt due to irreversible proprioceptive Piezo2 channelopathy in ALS [40].

**Figure 1:**
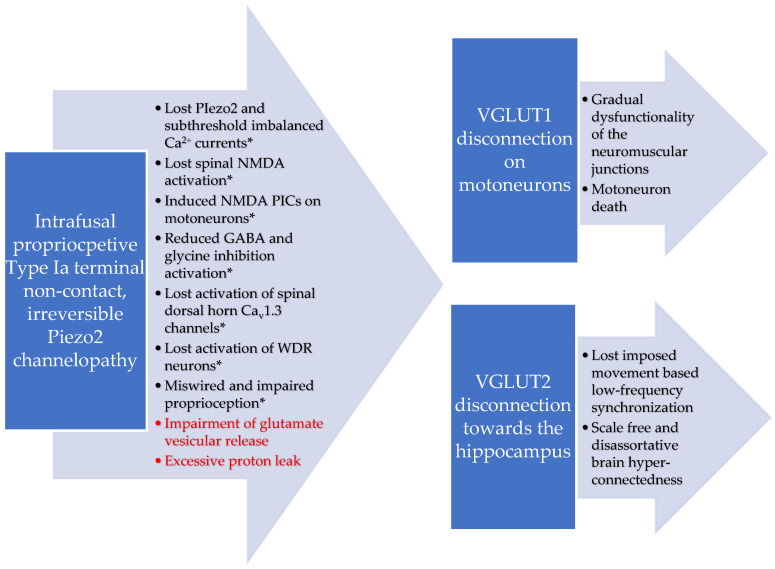
Proposed VGLUT 1 and VGLUT2 disconnections and resultant dysregulated proton handling due to the theorized irreversible Piezo2 channelopathy in ALS. * Adapted from previously published figure [14].

Furthermore, high lactate is not favorable for the survival of NKT cells, in contrast to the effect of glutamine [68]. Lactate shuttle releases glutamine into the extracellular space, and it is taken up by neurons [69,70,71]. However, this proposed malfunctional lactate shuttle machinery in conjunction with impaired glutamate vesicular release in DOMS could increase glutamine presence in the extracellular space [54]. As a result, this metabolic-switch-derived excess glutamine might also attract NKT cells due to their distinct metabolic programing [68]. Moreover, the proton-based ultrafast long-range VGLUT2 signaling that may bridge the intrafusal space to the hippocampus during voluntary exercise could also be impaired [19]. This is in addition to the proposed VGLUT1/Ia synaptic disconnection on motoneurons [14,18]. The progressive loss of synchronization and the grading/scaling of this proton-based ultrafast signaling in ALS could be one explanation (see Figure 1) for the observed phenomenon of scale-free and disassortative hyper-connectedness as the disease progresses [72].

It is noteworthy that a role of Piezo2 in the synchronization of peripheral proprioceptive input to brain rhythms, like theta rhythms, and the grading of proton-based ultrafast signaling has been hypothesized lately [19]. This new theory is in line with the earlier coupled oscillator model, namely, when the oscillator(s) in the central nervous system is entrained to the imposed forcing peripheral oscillator [73]. Furthermore, the study also found that the likely source of this imposed movement-based low-frequency forcing peripheral oscillator is the Ia afferent signal from the muscle spindle [73]. Therefore, the theorized loss of proprioceptive terminal Piezo2 function in ALS may connect proton-based ultrafast signaling to dissimilar properties within brain networks in a hyper-connected manner, because the imposed movement-based forcing peripheral oscillator is non-functioning (see Table 1). It is noteworthy that functional Piezo2 is proposed to act like a low-frequency Shottky barrier semiconductor diode [19]. Consequently, the author also suggests that Piezo2 and ASIC2 co-signaling is indeed needed for proprioceptive signaling in agreement with Bornstein et al. [49]. However, rather, Piezo2 could be the ion channel providing the grading and scaling for ASIC2 proton handling towards the proposed proton-based ultrafast long-range signaling through VGLUT1 and VGLUT2 to motoneurons and hippocampus, respectively.

An interesting recent study reveals hippocampal metabolic alterations in ALS [74]. Indeed, evidence of hippocampal involvement and degeneration is emerging in ALS [75,76]. These hippocampal metabolic alterations are especially interesting in light of a new theory postulating that atypical hippocampal-like metabotropic glutamate receptors coupled to phospholipase D (PLD-mGluR) containining intrafusal proprioceptive primary afferents with activated Piezo2 on their terminals may synchronize to theta rhythm ON [19]. This long-range synchronization is likely induced by hippocampal medial septal glutamatergic neurons with the assistance of hippocampal Piezo2 [19]. It is important to note again that Piezo2 functions like a low-frequency Shottky barrier semiconductor diode in this mechanism [19]. Moreover, this theory also proposes that this novel long-range ultrafast resonance-based cross-frequency coupled signaling could provide peripheral, spatial, and speed inputs to the space and speed coding of hippocampal theta rhythm oscillations [19]. These inputs likely support locomotion and episodic memory: more importantly, spatial memory [19]. It is noteworthy that syndecan-3 may have a role in hippocampal synaptic plasticity and its deficiency impairs hippocampus-dependent spatial learning and memory [23], not to mention locomotion [77].

In addition, this atypical hippocampal-like metabotropic PLD-mGluR is homomeric to metabotropic GluK2 [37], and GluK2 has a role in the maintenance of glucose homeostasis [78]. Correspondingly, mGluRs and GABA are involved in glucose-stimulated insulin secretion from the beta cells of pancreas [79] and Piezo1 plays a critical role in this mechanism [80]. In the meantime, syndecan-3 transduces external cell surface signals intracellularly through the actin cytoskeleton in order to participate in cell shape organization and this process is sugar-dependent [81]. Moreover, a recent pilot study found that DOMS-inducing fatiguing exercise, suggested to involve Piezo2 channelopathy as the primary injury phase [18,26], resulted in reduced orthostatic tolerance with associated abnormal changes in diastolic blood pressure and heart rate in a similar fashion to in diabetes mellitus [82].

The author of this manuscript suggests that the progressive irreversible intrafusal Piezo2 channelopathy in ALS diminishes the grading and scaling of peripheral synchronization into hippocampal theta rhythms as the disease progresses. This altered synchronization possibly leads to the observed hippocampal metabolic alterations, hippocampal volume reduction, and spatial memory impairment in ALS. This theoretical proprioceptive Piezo2 channelopathy-derived hippocampal metabolic alteration may eventually lead to a more widespread metabolic dysfunction that propels the ALS disease course. This may include progressive acidosis, glycogen accumulation, and lipolysis [83]. It is notable that voluntary exercise enhances spatial memory [84] and this finding is in support of the theory that Piezo2-induced enhanced resonance initiates the synchronization of the theta rhythm ON by providing the spatial input into the hippocampal theta rhythm [19]. Spatial memory encoding involves syndecan-3 [23] and Piezo1 [85]. DOMS may interfere with this hippocampal theta rhythm synchronization due to acute Piezo2 channelopathy and functional syndecan-3 depletion. In contrast, the loss of proprioceptive Piezo2 activity and resultant lost Piezo2–Piezo1 crosstalk and chronic syndecan functional depletion could contribute to impaired spatial memory in ALS. This reduced spatial memory dimension of ALS seems not to largely affect cognition in a constrained environment but may cause unattainable challenges in unfamiliar or complex environments.

It was suggested earlier that the chronic Piezo2 channelopathy-induced impaired Piezo2–Piezo1 crosstalk will deplete certain proteoglycans on the chronic path [86]. Even more importantly syndecans are suspected in this mechanotransduction-associated depletory mechanism [17] in the aforementioned manner. Interestingly, chronic-stress-induced muscle acidification leading to muscle mechanical hyperalgesia involves extracellular matrix proteoglycans and ASIC3 [87]. The implicated versican seems to partially compensate for the suggested lost function of upstream syndecans. Indeed, ASIC3 contributes to proprioceptive mechanotransduction in a secondary manner, especially in the case of the theorized chronic Piezo2 channelopathy [18,27]. However, this compensatory mechanism is suggested to be absent in the later, painless symptomatic phase of ALS [13] due to the irreversible stage of Piezo2 channelopathy [41]. Accordingly, the upregulation of versican could be observed in the early symptomatic stage of ALS, but depletion could be observed in the late symptomatic stage [88]. Moreover, a significant upregulation of ASIC2 and ASIC3 was reported in the motor neurons and spinal cord of the SOD1 mutant of ALS patients [47,89].

Excessively prolonged mechanotransductions on Piezo2 channels may induce shedding on syndecans-3, as noted before, which may alter its negative charge on the cellular surface. Therefore, syndecan-3 could contribute to the impairment of the Piezo2–Piezo1 crosstalk in the affected compartmental micromilieu. Consequently, adequate syndecan supply and unimpaired extracellular surface representation seem to be needed in neuron activation and regeneration. In support, synthetic proteoglycans have been capable of reversing neuron regeneration disruption [90], most likely due to the redundant features of proteoglycans and syndecans. However, the chronic Piezo2 channelopathy in combination with functional syndecan depletion and underlying genetic mutations could make Piezo2 channelopathy irreversible, and this could be an important pathomechanistic link in ALS.

## 4. Additional Likely Role of Snydecan-3 and MyoD in ALS

Syndecan-3 has functional relevance in satiety signaling in feeding control [91]. This signaling is activated by food intake and transduced by neurons from the gastrointestinal tract to the brain [92]. It is interesting to note that research is emerging that Piezo also has a functional role in gut mechanosensation by the volume-based control of meal size in feeding control signaling [93]. Moreover, the Piezo2 control of gastrointestinal transit in humans and mice has been also identified [94]. Unsurprisingly, gastrointestinal dysfunction is evident in ALS, where it takes the form of delayed gastric emptying and delayed colonic transit times [95]. Therefore, impaired feed control signaling may have a role in the ALS disease process, since the undesirable loss of appetite and early weight loss are ongoing challenges for these patients from onset to diagnosis [96,97,98].

The recognized involvement of syndecan-3 in inflammation control [21] is intriguing in light of a recent study. This study implies that the acute proprioceptive terminal Piezo2 channelopathy may induce the inflammatory reflex [17]. Furthermore, the chronic Piezo2 channelopathy of these neurons may induce the gateway reflex as the chronification of the inflammatory reflex [17]. Syndecan-3 may bear a central role in these reflexes. Correspondingly, dendritic cells within the Piezo2 microinjured compartmental micromilieu are activated with Piezo1 downregulation as a consequence of the impaired Piezo2–Piezo1 crosstalk [19]. Myocytes are also activated in this process, but with Piezo1 upregulation [19]. In parallel, Piezo2 channelopathy also activates the first-line innate immune system with the active involvement of syndecans, leading to the aforementioned imbalanced control of NKT cells [19,40]. Furthermore, Piezo1 participates concomitantly in the activation of satellite cells [67] within these proprioceptive Piezo2 microinjured compartments. Interestingly, not only does Piezo1 participate in the satellite cell activation and muscle regeneration process [67] of this inflammatory reflex mechanism, but syndecan-3 does, as well [99]. It is a noteworthy consideration that this inflammatory reflex mechanism is a bi-phasic and bi-compartmental intra-extrafusal interplay, like in DOMS [18], when the selective barriers of the affected compartments become more permeable due to the primary Piezo2 microdamage-induced impaired Piezo2–Piezo1 crosstalk [17].

Angiogenesis is an integral part of this regenerative mechanism and intrinsically coupled to inflammation where syndecane-3 has a central role [21]. Not surprisingly, Piezo1 also mediates angiogenesis [100]. Indeed, syndecan-3 has an essential role in skeletal muscle regeneration [77] and remodeling. Syndecan-3 knock-out mice exhibit hallmark muscular dystrophy in association with impaired locomotion, fibrosis, and hyperplasia of myonuclei and satellite cells by mislocalizing MyoD that leads to altered differentiation [77]. The presentation of this MyoD-involved signaling pathway is significant, considering the early regeneration process, when the initial activation of satellite cells takes place [77]. MyoD is, rather, presented in fast-twitch muscle fibers, in contrast to slow-twitch fibers [101]. It is noteworthy that MyoD gene transfer worsens survival and propels motoneuron degeneration and the denervation of affected muscles in an ALS mouse model [102]. The relevance of the differential expression pattern of MyoD is rightly emphasized, as the metabolic shift to oxidative metabolism underpins the switch from fast- to slow-twitch muscle in ALS disease progression [103]. This switch is meant to sustain muscle function and postural control. It is worth mentioning that the underlying muscle-spindle-derived proprioceptive neural switch mechanism was theorized earlier [13]. Respectively, DOMS is the acute transient and ALS is the chronic irreversible phenomena of this proposed proprioceptive distal axonopathy [13]. Even more importantly, this theoretical proprioceptive terminal mechano-energetic microinjury-induced switch was demonstrated to delay the medium latency response of the stretch reflex in DOMS [51]. Correspondingly, this primary damage was named as an acquired acute transient Piezo2 channelopathy in DOMS [18,26] and an irreversible one in ALS [14,41] (see Table 1). Even the underlying metabolic switch and mitochondrial dysfunction have been implicated in the proprioceptive terminal Piezo2 channelopathy theory [14,19,26,40]. In support, a recent finding demonstrated that MyoD family inhibitor proteins, MDFIC and MDFI, are indeed auxiliary subunits of Piezo1 and Piezo2 ion channels and they participate in the control of Piezo inactivation [104]. Moreover, the absence of syndecan-3 significantly increases protein phosphorylation [77]. The current author proposes that syndecan-3 shedding and functional depletion under acute or chronic allostasis could contribute to the aforementioned metabolic switch through phosphorylation in acute reversible and irreversible Piezo2 channelopathy, respectively. Most likely, the excessively prolonged mechanotransduction of proprioceptors causes a metabolic switch in the satellite cells of the lactate shuttle machinery. Eventually, this impaired lactate shuttle mechanism during excessively prolonged mechanotransduction and concomitant allostasis evolves into the Piezo2 channelopathy. Furthermore, the transient or irreversible (may be functional) loss of the auxiliary connection of MyoD family inhibitor proteins to Piezo ion channels may contribute to the acquired acute reversible Piezo2 channelopathy and the irreversible one, correspondingly. Since MyoD is a transcription factor, the (functional) loss of the auxiliary connection of MyoD family inhibitor proteins could represent one explanation of the proposed principal transcription activator function of Piezo2 channelopathy [14]. This is indicative that muscle-injured syndecan-3 knock-out and wild-type mice could not bear weight on the injured leg for 2–3 days after injury [77]. It is noteworthy that this time window overlaps with the ascending phase of mechanical hyperalgesia in DOMS [30] and the proposed Peizo2 channelopathy [26]. Piezo2 channelopathy is suggested to be one principal gateway between physiology and pathophysiology [27] and the gateway to remodeling [19]. Therefore, it is not surprising that skeletal muscle remodeling is considered as a function of disease progression in ALS [105]. However, it is important to note again that these mechanisms are severely and progressively impaired due to the theorized irreversibility of Piezo2 channelopathy and resultant lost crosstalk between Piezo2 and Piezo1 in the affected compartments of ALS.

## 5. Conclusions

This paper theorizes that shedding and charge altering variants of the syndecan-3-encoding SDC3 gene may play a critical role in the ALS pathomechanism as a result of irreversible Piezo2 channelopathy. Accordingly, this acquired irreversible Piezo2 microinjury may be the primary damage that could not only reveal the underlying genetic and environmental risk factors in ALS, but could also disrupt the Piezo2–Piezo1 crosstalk. Furthermore, it could also disrupt the proton-based ultrafast long-range proprioceptive signaling toward the motoneurons and hippocampus through VGLUT1 and VGLUT2, respectively (see Figure 1).

Syndecan-3 is likely to play a central mediatory role in the Piezo2–Piezo1 crosstalk. Accordingly, excessively prolonged mechanotransduction under allostasis could bear relevance in the evolvement of Piezo2 channelopathy due to the altered cell surface charge of syndecan-3 by shedding or charge-atltering variants of the SDC3 gene. This altered charge may also sustain the impaired Piezo2–Piezo1 crosstalk. Moreover, the charge-altering variants of syndecan-3-encoding SDC3 gene may have special relevance to the disruption of this crosstalk during the aging process, since Piezo2 channelopathy is theorized to be also subject to mitochondrial dysfunctionality [14,26]. Consequently, the suggested syndecan-3-related mechanisms in association with the proposed irreversible Piezo2 channelopathy may provide one explanation for the onset of the ALS pathomechanism.

Finally, an inert proton-based long-range ultrafast signaled proprioceptive skeletal system is emerging that is suggested to be gradually lost in ALS due to the theorized progressive irreversible Piezo2 channelopathy. This may explain how soft and exoskeletal robotic-assisted therapeutic exercises show preventive or even positive results in ALS [106,107,108,109]. Correspondingly, the author proposes that the dying mechanism in ALS is backwards if it is looked at from a somatosensory angle. On the contrary, it is propelled forward when it is considered from a motoneuronal angle. However, research is emerging that sensory dysfunction comes earlier in ALS. Therefore, ALS is suggested to be initiated by a dying-back mechanism, regardless of it being considered a lethal motor neuron disease.

Paired associative treatment with combined transcranial and peripheral electromagnetic stimulation seems to compensate for the aforementioned lost inert proton-based long-range ultrafast proprioceptive signaling on motoneurons and toward the hippocampus. This could be the case in DOMS, as well, where only this combined treatment was demonstrated to be a promising accelerated healing method [110]. Indeed, peripheral neuromuscular magnetic stimulation was shown to combat the waning of muscles in ALS [111], as transcranial magnetic stimulation has positive effects [112] and not only diagnostic utility [113]. Indeed, magnetic field stimulation brought back axonal organelle motility in cultured FUS-ALS motoneurons with signs of regeneration [114]. Thus, paired associative transcranial and peripheral electromagnetic stimulation could be a promising treatment method and not only a tool to research NMDA-mediated neocortical networks in ALS [115].

## 6. Limitations

One limitation of the aforementioned theories is that no in vitro and in vivo models of sporadic ALS exist yet to provide important support to validate the onset of the ALS pathomechanism specifically due to the theorized irreversible Piezo2 channelopathy. Motor-neuron-like NSC34 cells would be one in vitro model option [116]. However, these cells were demonstrated to be unsuitable as an experimental model for glutamate-mediated excitotoxicity [117]. However, it is noteworthy that the exposure to methylmercury exerts its neurotoxicity in ALS through the overexpression of Restrictive Element 1 Silencing Transcription factor (REST) [116] and Piezo2 channelopathy is proposed to be a principal transcription activator [14]. Most likely, Piezo1 channelopathy is also a transcription activator, and it is also important to note that Piezo1 channelopathy could also induce Piezo2 channelopathy under prolonged stress-induced mechanotransduction [27]. Another in vitro model, in which Resveratrol is capable of preventing thimerosal-induced cell death by Sirtuin 1 (SIRT1) activation [118], is indicative of Piezo1 involvement in the ALS pathomechanism, since Piezo1 could mediate the SIRT1-HIF1α glucose metabolism pathway [119]. The relevance of the addition of in vitro and in vivo models and mechanisms was emphasized in an earlier article [14] and revisited in a review [120]; therefore they were not a direct subject of this paper.

## Figures and Tables

**Table 1:** The updated table for the proposed primary damage mechanism in ALS and DOMS * [14,18,54].

	Amyotrophic Lateral Sclerosis (ALS)		Delayed-Onset Muscle Soreness (DOMS)	
**Primary intrafusal damage phase**	YES	Irreversible intrafusal proprioceptive terminal microdamage	YES	Transient intrafusal proprioceptive terminal microdamage
	YES	Irreversibly lost imposed movement-based low-frequency forcing oscillation from the Type Ia afferent signal due to irreversible Piezo2 channelopathy **	YES	Transiently lost imposed movement-based low-frequency forcing oscillation from the Type Ia afferent signal due to transient Piezo2 channelopathy **
	NO	Lost NMDA receptor activation on spinal dorsal horn due to irreversibly lost glutamate vesicular release on type Ia proprioceptive neurons	YES	NMDA receptor activation on spinal dorsal horn due to impairment of the glutamate vesicular release on type Ia proprioceptive neurons
	NO	Lost L-type calcium currents and nonspecific cationic currents in spinal dorsal horn due to lost Piezo2 functional at peripheral proprioceptive terminals	YES	L-type calcium currents and nonspecific cationic currents in spinal dorsal horn due to peripheral proprioceptive terminal Piezo2 channelopathy induced subthreshold imbalanced calcium currents
	NO	Wide dynamic range (WDR) neuron activation	YES	WDR neuron activation
**Soreness condition**	Considered as a painless disease		DOMS lasting up to 7 days	

* Both ALS and DOMS are suggested to contain intrafusal microdamage at proprioceptive terminals. However, in the case of ALS, it is proposed to be irreversible. On the contrary, the microdamage on proprioceptive terminals is transient in the case of DOMS. Hence, the progressive loss of intrafusal proprioceptive Piezo2 function will lead to neurodegeneration and eventually to death in ALS [14]. ** new mechanisms added to previously published table [14].

## Data Availability

Not applicable.
